# In vitro evaluation of microbial contamination and the disinfecting efficacy of chlorhexidine on orthodontic brackets

**DOI:** 10.1186/s40510-019-0270-4

**Published:** 2019-05-01

**Authors:** P. R. Vivek Aithal, K. R. Akshai Shetty, M. R. Dinesh, B. C. Amarnath, C. S. Prashanth, Mathew David Roopak

**Affiliations:** DAPM RV Dental College, Bengaluru, Karnataka 560078 India

**Keywords:** Contamination, Disinfection, Microbiology, Orthodontic brackets, Product packaging

## Abstract

**Background:**

Contamination of orthodontic appliances is due to the unhygienic practices followed during manufacturing and packaging processes, which may lead to cross-contamination. Although literature has indicated the need for sterilization or disinfection of orthodontic appliances before using in the oral cavity, this is still not employed in routine clinical practice. In this view, the current study evaluates the bacterial load on orthodontic brackets along with the disinfecting efficacy of chlorhexidine.

**Methods:**

A total of 140 brackets were obtained from four different manufacturers and divided into six groups: group 1 (American Orthodontics; *n* = 30), group 2 (3M Unitek; *n* = 30), group 3 (Ortho Organizers; *n* = 30), group 4 (China Dental Orthodontic; *n* = 30), group 5 (negative control; *n* = 10), and group 6 (positive control; *n* = 10). Various microbiological and biochemical tests were conducted on the brackets to detect the type and growth of bacteria. Brackets that showed microbial contamination were then subjected to disinfection using 0.01% and 2% chlorhexidine solutions.

**Results:**

Microbial contamination was detected on brackets of all the four groups. Bacteria, including *Staphylococcus aureus*, *S. epidermidis*, Lactobacilli, *Klebsiella pneumoniae*, *Bacillus licheniformis*, and *B. cereus*, were identified in these groups. Upon disinfection with 0.01% chlorhexidine solution, brackets in group 2 displayed complete decontamination, while all brackets in the other groups containing gram-negative bacteria exhibited complete decontamination with 2% chlorhexidine.

**Conclusion:**

Orthodontic brackets received from four manufacturers showed high bacterial contamination. Disinfecting ability of 2% chlorhexidine proved highly effective in destroying both gram-positive and gram-negative bacteria. Therefore, use of 2% chlorhexidine in clinical practice for the disinfection of orthodontic brackets is suggested, before placement in the oral cavity.

## Background

The human oral cavity contains several distinct microbial habitats, such as the teeth, the cheek, the lips, the tongue, the gingiva, the gingival sulcus, and the hard and soft palate [1, 2]. These habitats that act as reservoirs for several pathogenic organisms cause systemic infection and increase the risk of cross-contamination [3, 4]. Introduction of fixed or removable orthodontic appliances in the oral cavity may cause specific variations in the oral microflora by decreasing the pH, increasing the dental plaque accumulation, and raising the microbial count in saliva. Further, these changes contribute to the increased risk of cross-contamination [3]. In addition, infection in the oral cavity might also be due to the use of contaminated instruments or straight use of orthodontic appliances received from the manufacturer’s packaging without disinfection [5].

Pathogens involved in transmitting the infection include viruses, such as hepatitis B and C, herpes simplex, and human immunodeficiency. Further, bacterial contaminations due to *Mycobacterium tuberculosis*, Staphylococcal and Streptococcal spp., and other microorganisms are responsible for the upper respiratory tract infections [6]. Among all of these, gram-positive Staphylococci are considered as the major cause of nosocomial infections. [7]

Heat sterilization and disinfection are the effective methods to eliminate microorganisms causing contamination. However, literature has reported chemical disinfection to be more effective in reducing contamination when compared to heat sterilization [8]. Glutaraldehyde, hydrogen peroxide, alcohol, and chlorhexidine are the disinfectants commonly used in the chemical sterilization process [8, 9]. Currently, chlorhexidine is the most favorable disinfectant due to its broad-spectrum bactericidal action against both the gram-positive and gram-negative bacteria [8].

Several in vitro and in vivo studies reported microbial contamination in orthodontic appliances received directly from the manufacturers [1, 10, 11]. However, data regarding sterilization protocol and use of disinfecting agents to overcome the bacterial contamination are lacking in the literature. Although the instruments used in dental practice are adequately sterilized, this is not factual for orthodontic appliances, such as brackets, bands, and archwires. As a responsible clinician, the objective is to break the circle of infection by avoiding contamination. Based on these observations, the present study was conducted to evaluate the bacterial load of orthodontic brackets received from manufacturers and to assess the efficacy of in vitro disinfectant—chlorhexidine—on the contaminated orthodontic brackets.

## Methods

**Fig. 1:**
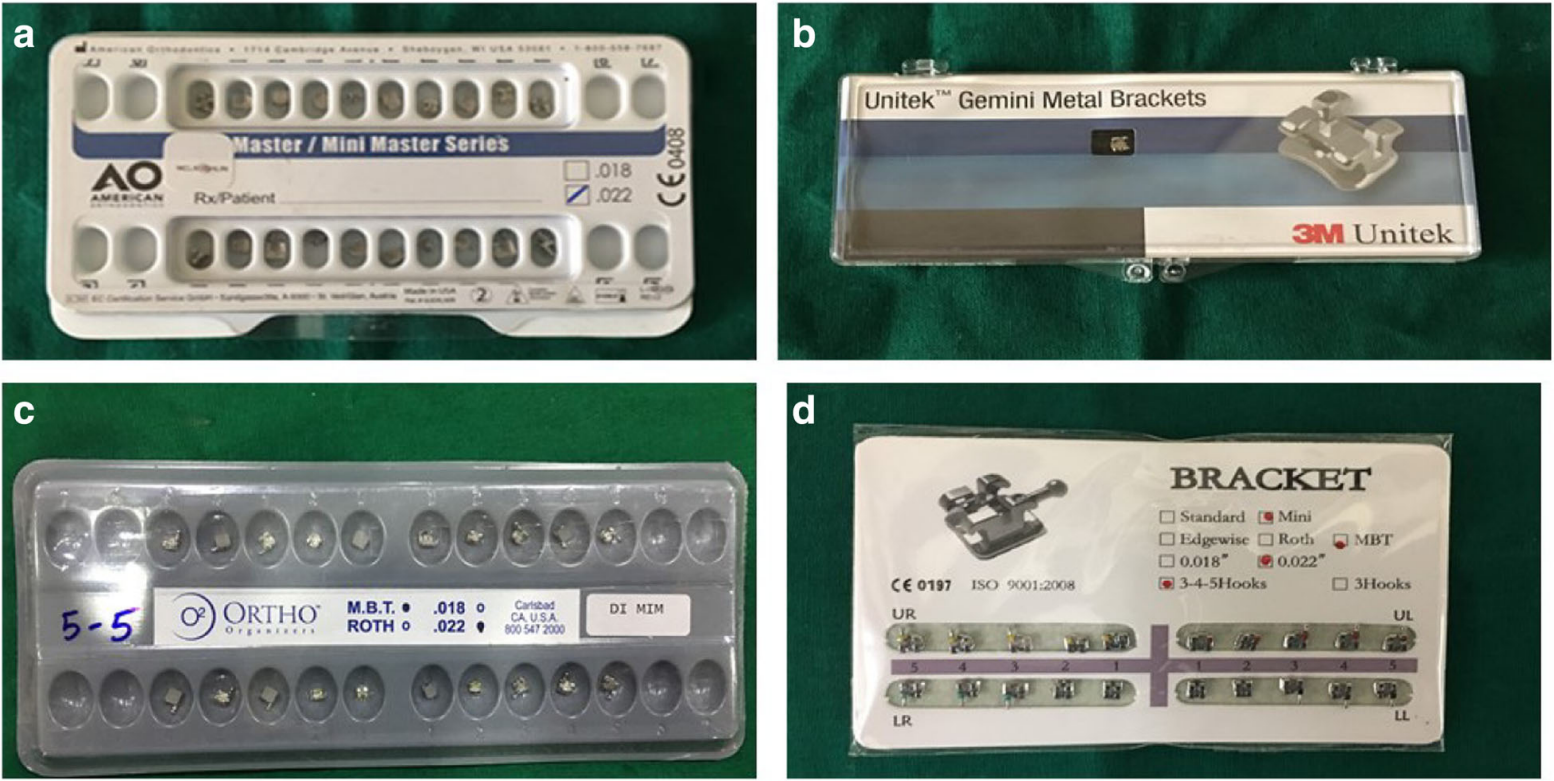
Orthodontic bracket kits. **a** American Orthodontics brackets. **b** 3M Unitek. **c** Ortho Organizers. **d** China Dental Orthodontic Brackets

The in vitro microbiological and biochemical investigations in the present study were conducted at the Department of Microbiology. The sample consisted of 140 intact orthodontic bracket kits received from four different manufacturers. Molar buccal tubes and damaged/tampered orthodontic bracket kits were not included in the analysis. All the 140 brackets were divided into six groups with groups 1 to 4 consisting of 30 brackets each and groups 5 and 6 consisting of 10 brackets each. The manufacturers included American Orthodontics (group 1), 3M Unitek (group 2), Ortho Organizers (group 3), and China Dental Orthodontic Brackets (group 4) with 30 bracket samples each (Fig. 1). Group 5 (negative control group) consisted of American Orthodontics brackets that were sterilized in surgical grade paper in an autoclave to confirm the absence of bacterial growth, whereas group 6 (positive control group) consisted of 3M Unitek brackets that were contaminated with *S. aureus* to determine the maximum bacterial growth.

All the samples were subjected to microbiological tests to determine the presence of bacterial growth and biochemical tests to identify the type of bacteria.

### Microbiological tests

**Fig. 2:**
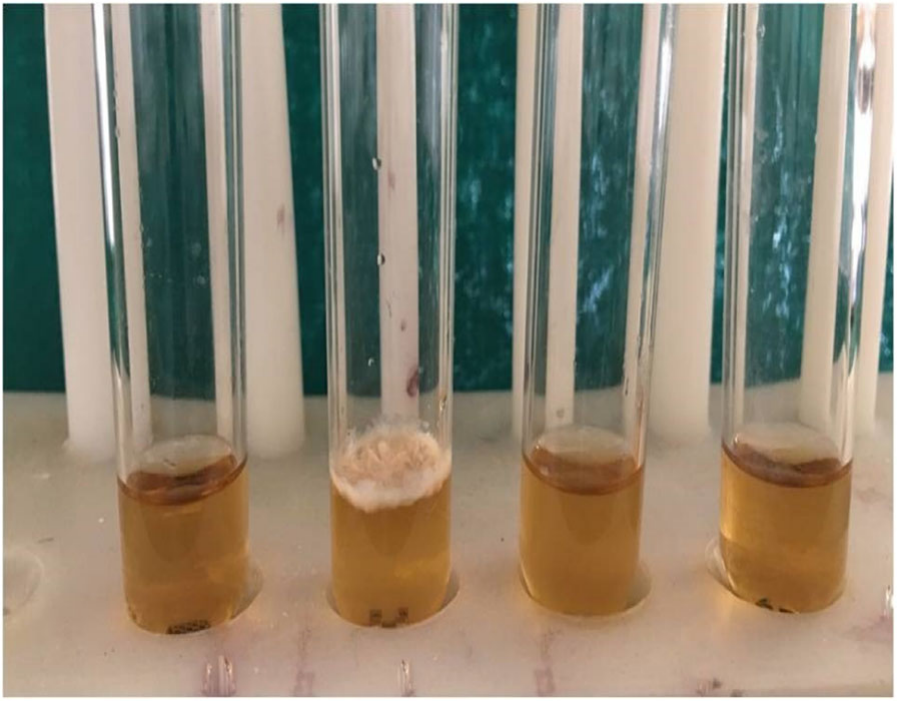
One of the brackets (2nd tube from the left) showing microbial growth in the brain heart infusion broth

Orthodontic brackets in all the groups were immersed individually in test tubes containing 3 ml of sterilized brain heart infusion (BHI) broth and placed in an incubator for 48 h at 35 °C to evaluate the bacterial growth. The bacterial growth was assessed based on changes in the color/turbidity of the medium in each of the tubes (Fig. 2). The tubes that were positive for bacterial growth were further subjected to biochemical analysis.

### Biochemical tests

Biochemical analysis was performed for the tubes that exhibited bacterial growth. The organisms were grown on blood agar medium using a streak plate technique. Later, the blood agar plates were incubated at 35 °C for 48 h, and the plates displaying growth of colonies were subjected to the gram staining protocol. The colonies were observed under a microscope to differentiate between gram-positive and gram-negative bacteria. Based on the morphological characteristics of the bacteria in each sample, they were subjected to biochemical tests for identification. Catalase test, citrate utilization test, coagulase test, indole test, lactose fermentation test, and oxidase test were performed to identify different gram-positive and gram-negative isolates involved in the contamination of the brackets.

### Disinfection

The contaminated brackets were subjected to disinfection individually using 0.01% chlorhexidine solution for 5 min (phase I decontamination). Later, all the brackets were removed from the solution with sterile pincers and dried with oil-free compressed air for 60 s. Microbiological test was conducted again on all the brackets to assess the efficiency of 0.01% chlorhexidine solution. The brackets that displayed contamination after the first disinfection were again subjected to a higher concentration of chlorhexidine (2%) and assessed for its efficiency (phase II decontamination).

### Statistical analysis

SPSS 22.0 (IBM Corp, Armonk, NY, USA) was used to analyze the data. Descriptive analysis of all the study parameters was done using frequency and proportions. A chi-square test was used to compare the microbial contamination between different study groups at different time intervals. Cochran’s Q test and McNemar’s test were used to assess the microbial presence between different time intervals in each study group. *P* < 0.05 was considered statistically significant.

## Results

A statistically significant variation in microbial contaminations was observed among all the groups (*P* < 0.001) at baseline. The highest number of brackets was contaminated in group 4, whereas the least contamination was observed in group 2 (Table 1).

**Table 1:** Comparison of microbial contamination between the different study groups at the baseline phase

Groups	Microbial contamination		*P* value
	Absent, *n* (%)	Present, *n* (%)	
Group 1	17 (56.7)	13 (43.3)	< 0.001^*^
Group 2	23 (76.7)	7 (23.3)	
Group 3	13 (43.3)	17 (56.7)	
Group 4	6 (20.0)	24 (80.0)	
Group 5	10 (100.0)	0	
Group 6	0	10 (100.0)	

*Group 1*, American Orthodontics brackets; *Group 2*, 3M Unitek; *Group 3*, Ortho Organizers; *Group 4*, China Dental Orthodontic Brackets; *Group 5*, negative control; *Group 6*, positive control

^*^Statistically significant at 95% confidence interval

According to the results of the microbiological tests, none of the samples in group 5 showed darkening/turbidity of the BHI medium, confirming the absence of bacterial growth. In contrast, all specimens in group 6 showed a darkened medium, suggestive of bacterial growth.

The microbial contamination in group 1 was not associated significantly with groups 2 and 3; similarly, groups 2 and 4 were not associated significantly with groups 5 and 6, respectively. However, other pairs showed a statistically significant difference in the microbial contamination (Table 2).

**Table 2:** Pairwise comparison of microbial contamination between the groups

Groups		*P* value
G1	G2	0.10
	G3	0.30
	G4	0.003^*^
	G5	0.01^*^
	G6	0.002^*^
G2	G3	0.008^*^
	G4	< 0.001^*^
	G5	0.09
	G6	< 0.001^*^
G3	G4	0.05^*^
	G5	0.002^*^
	G6	0.01^*^
G4	G5	< 0.001^*^
	G6	0.13
G5	G6	< 0.001^*^

*G1*, American Orthodontics brackets; *G2*, 3M Unitek; *G3*, Ortho Organizers; *G4*, China Dental Orthodontic Brackets; *G5*, negative control; *G6*, positive control

^*^Statistically significant at 95% confidence interval

The microorganisms isolated in group 4 were higher compared to those in groups 1, 2, and 3. The most predominant microorganisms isolated from groups 3 and 4 were *Staphylococcus epidermidis* and *Klebsiella pneumoniae*, respectively. *S. epidermidis* and *Bacillus cereus* were equally present in group 1, whereas non-pathogenic bacteria were only observed in group 2 (Table 3).

**Table 3:** Organisms isolated from the different groups

Organisms	Group 1, *n* (%)	Group 2, *n* (%)	Group 3, *n* (%)	Group 4, *n* (%)
*Staphylococcus aureus*	1 (7.69)	–	5 (29.41)	3 (12.5)
*S. epidermidis*	3 (23.08)	2 (28.57)	7 (41.18)	5 (20.83)
*Lactobacilli*	1 (7.69)	–	1 (5.88)	3 (12.5)
*Klebsiella pneumoniae*	3 (23.08)	–	1 (5.88)	7 (29.17)
*Bacillus licheniformis*	2 (15.38)	–	2 (11.76)	3 (12.5)
*B. cereus*	3 (23.08)	1 (14.29)	1 (5.88)	3 (12.5)
Non-pathogenic bacteria	–	4 (57.14)	–	–

*Group 1*, American Orthodontics brackets; *Group 2*, 3M Unitek; *Group 3*, Ortho Organizers; *Group 4*, China Dental Orthodontic Brackets

After phase I decontamination with 0.01% chlorhexidine solution, group 2 showed complete disinfection, whereas group 4 showed the highest microbial contamination followed by groups 1 and 3. However, no significant association was observed between phase I decontamination and microbial presence on orthodontic brackets (*P* = 0.22). All the groups were completely disinfected after phase II decontamination with 2% chlorhexidine solution (Table 4).

**Table 4:** Association of microbial occurrence in different groups with phase I and II decontamination

Groups	Absent, *n* (%)	Present, *n* (%)	*P* value
Phase I decontamination			
Group 1	10 (76.9)	3 (23.1)	0.22
Group 2	7 (100)	0	
Group 3	16 (94.1)	1 (5.9)	
Group 4	18 (75)	6 (25)	
Phase II decontamination			
Group 1	3 (100)	0	–
Group 3	1 (100)	0	
Group 4	6 (100)	0	

*Group 1*, American Orthodontics brackets; *Group 2*, 3M Unitek; *Group 3*, Ortho Organizers; *Group 4*, China Dental Orthodontic Brackets

After phase I decontamination, the microbial load in group 2 was decreased to a statistically significant degree (*P* = 0.05), while the microbial load in groups 1, 3, and 4 was significantly decreased after phase II decontamination (Table 5).

**Table 5:** Comparison of microbial presence between the baseline and different phases of decontamination

Groups	Time	Absent, *n* (%)	Present, *n* (%)	*P* value
Group 1^	Baseline	17 (56.7)	13 (43.3)	0.02*
	Phase I	10 (76.9)	3 (23.1)	
	Phase II	3 (100.0)	0	
Group 2#	Baseline	23 (76.7)	7 (23.3)	0.05*
	Phase I	7 (100.0)	0	
Group 3^	Baseline	13 (43.3)	17 (56.7)	0.01*
	Phase I	16 (94.1)	1 (5.9)	
	Phase II	1 (100.0)	0	
Group 4^	Baseline	6 (20.0)	24 (80.0)	0.002*
	Phase I	18 (75.0)	6 (25.0)	
	Phase II	6 (100.0)	0	

*Group 1*, American Orthodontics brackets; *Group 2*, 3M Unitek; *Group 3*, Ortho Organizers; *Group 4*, China Dental Orthodontic Brackets; *Group 5*, Negative control; *Group 6*, Positive control

*Statistically significant, ^Cochran’s *Q* test, ^#^McNemar’s test

## Discussion

Studies have suggested the need for sterilization or disinfection of materials prior to their administration in the oral cavity [8, 12]. However, the use of orthodontic appliances directly from the manufacturer’s packages is still a routine clinical practice. According to previous studies, orthodontic appliances received from the manufacturer’s packages were unsterile [1, 10, 11]. Therefore, the present study evaluated the bacterial load of the orthodontic brackets received from different manufacturers and determined the efficacy of chlorhexidine in destroying the microbial contamination.

Bacterial colonization was confirmed in all the orthodontic brackets received from different manufacturers. The outcome of the current investigation was similar to prior studies using different orthodontic appliances, such as arch wires [1], orthodontic pliers [6], brackets [11], orthodontic buccal tubes [4], and tooth brushes [13] received from different manufacturers. These studies indicate that orthodontic appliances used in dentistry are often contaminated with bacteria.

In our study, Staphylococci were the predominant organisms isolated from orthodontic brackets. Contamination with Staphylococci mostly occurs due to skin contact during manufacturing and/or packaging of orthodontic appliances [1, 6]. Similar studies conducted in this regard reported that Staphylococci were the common organisms to contaminate the orthodontic brackets [1, 3, 14]. In our study, *B. cereus* and *B. licheniformis* were the other frequently isolated organisms from the orthodontic brackets, followed by Streptococci. *Bacillus* spp. cause food-borne diseases as well as nosocomial outbreaks in immune-suppressed hospitalized patients [15].

*K. pneumoniae* is the respiratory pathogen that was isolated from orthodontic brackets in our study. The infection spreads from one person to the other through contaminated hands of individuals in the hospital. A similar study conducted by Rastogi et al. [10] isolated *Klebsiella* spp. from the orthodontic brackets. Further, literature reported a direct association of *Klebsiella* spp. with autoimmune disorders, such as ankylosing spondylitis, rheumatoid arthritis, and Crohn’s disease [16, 17]. Isolation of *Lactobacilli* spp. that initiate and progress dental caries/decay was relatively low in our study [18]. All these potential microorganisms are of major health concern; therefore, it is essential to sterilize or disinfect the brackets before fixing in the oral cavity. The other non-pathogenic bacteria isolated from the brackets in group 2 were not detrimental to the patients’ health.

Chlorhexidine used in various medical fields, such as gynecology, urology, and ophthalmology, has a broad antimicrobial activity [19]. Several studies demonstrated that chlorhexidine is effective both as an antiplaque and antimicrobial agent. Depending on different concentrations, it has both bacteriostatic and bactericidal properties [19, 20]. Research has further reported that chlorhexidine does not affect the shear bond strength of orthodontic brackets and clinically exhibits acceptable bond strength [20]. Speer et al. also reported that chlorhexidine did not affect the bond strength of metal brackets; however, it reduced the bond strength of ceramic brackets [21]. In our study, two concentrations (0.01% and 2%) of chlorhexidine were used to disinfect the orthodontic brackets received from different manufacturers. Initially, the most commonly used 0.01% chlorhexidine, commercially available as mouthwash, was used for disinfection. However, complete disinfection was not observed in all the groups. Due to incomplete disinfection, 2% chlorhexidine solution—the next higher concentration used in the medical field—was used for disinfection. The exact mechanism exerted by chlorhexidine in destroying the bacteria is not yet clear [22]. However, it has been postulated that positively charged chlorhexidine molecules bind to the negatively charged lipid molecules of the cell membrane and interfere with the process of osmosis. [22] The other novel approach that can be used to reduce the bacterial contamination of orthodontic brackets is application of antimicrobial nanoparticles [23]. The different methods include coating of orthodontic brackets with a thin film of nitrogen-doped titania nanoparticles; combination of glass ionomer or resin-modified glass ionomer cements with fluorapatite, fluorohydroxyapatite, or hydroxyapatite nanoparticles; addition of titania, silica, or silver nanoparticles to acrylic orthodontic materials; and incorporation of nanofillers or silica/titania nanoparticles into orthodontic adhesives [23].

Studies have demonstrated that slightly higher concentrations of chlorhexidine are required to kill gram-negative pathogens than those required to kill the gram-positive pathogens [24, 25]. Due to the presence of a permeable cell wall in the gram-positive bacteria, they are destroyed easily when compared to the gram-negative bacteria [26]. Organisms present in group 2 were gram-positive and non-pathogenic bacteria. Therefore, a lower concentration (0.01%) of chlorohexidine was adequate to destroy all the bacteria. However, both gram-positive and gram-negative bacteria were observed on brackets in other groups, which required a higher concentration (2%) of chlorhexidine for complete decontamination.

Although unique, the current study has some potential limitations. As the study was conducted in in vitro conditions, further in vivo studies are required to support these findings. While orthodontic brackets showed complete decontamination after treatment with 2% chlorhexidine, there is no data related to long-term effectiveness of chlorhexidine to impede the growth of microorganisms.

Overall, the results advocate that the orthodontic brackets received from the manufacturer require suitable disinfection to safeguard the patients’ health. Furthermore, clinicians should be cautious about the use of contaminated appliances prior to administering in the oral cavity as it might affect the systemic health of the patients.

## Conclusion

Orthodontic brackets received from different manufacturers displayed high bacterial contamination. Disinfecting ability of 2% chlorhexidine proved highly effective in destroying both gram-positive and gram-negative bacteria. Therefore, it can be employed in clinical practice for the disinfection of orthodontic brackets. However, further in vivo clinical studies are required to validate our findings. It is also essential to practice the disinfection of orthodontic appliances to safeguard the patients’ systemic health.
